# Three Novel Rice Genes Closely Related to the *Arabidopsis* *IRX9*, *IRX9L*, and *IRX14* Genes and Their Roles in Xylan Biosynthesis

**DOI:** 10.3389/fpls.2013.00083

**Published:** 2013-04-10

**Authors:** Dawn Chiniquy, Patanjali Varanasi, Taeyun Oh, Jesper Harholt, Jacob Katnelson, Seema Singh, Manfred Auer, Blake Simmons, Paul D. Adams, Henrik V. Scheller, Pamela C. Ronald

**Affiliations:** ^1^Department of Plant Pathology, The Genome Center, University of CaliforniaDavis, CA, USA; ^2^Joint BioEnergy InstituteEmeryville, CA, USA; ^3^Sandia National LabsLivermore, CA, USA; ^4^Physical Biosciences Division, Lawrence Berkeley National LaboratoryBerkeley, CA, USA; ^5^Section for Plant Glycobiology, Department of Plant and Environmental Sciences, VKR Research Centre Pro-Active Plants, University of CopenhagenFrederiksberg C, Denmark; ^6^Life Sciences Division, Lawrence Berkeley National LaboratoryBerkeley, CA, USA; ^7^Department of Plant and Microbial Biology, University of CaliforniaBerkeley, CA, USA; ^8^Department of Plant Molecular Systems Biotechnology and Crop Biotech Institute, Kyung Hee UniversityYongin, Korea

**Keywords:** xylan, irregular xylan mutants, cell walls, type II cell walls, xylosyltransferase

## Abstract

Xylan is the second most abundant polysaccharide on Earth, and represents a major component of both dicot wood and the cell walls of grasses. Much knowledge has been gained from studies of xylan biosynthesis in the model plant, *Arabidopsis*. In particular, the irregular xylem (*irx*) mutants, named for their collapsed xylem cells, have been essential in gaining a greater understanding of the genes involved in xylan biosynthesis. In contrast, xylan biosynthesis in grass cell walls is poorly understood. We identified three rice genes *Os07g49370* (*OsIRX9*), *Os01g48440* (*OsIRX9L*), and *Os06g47340* (*OsIRX14*), from glycosyltransferase family 43 as putative orthologs to the putative β-1,4-xylan backbone elongating *Arabidopsis* *IRX9*, *IRX9L*, and *IRX14* genes, respectively. We demonstrate that the over-expression of the closely related rice genes, in full or partly complement the two well-characterized *Arabidopsis irregular xylem* *(irx)* mutants: *irx9* and *irx14*. Complementation was assessed by measuring dwarfed phenotypes, irregular xylem cells in stem cross sections, xylose content of stems, xylosyltransferase (XylT) activity of stems, and stem strength. The expression of *OsIRX9* in the *irx9* mutant resulted in XylT activity of stems that was over double that of wild type plants, and the stem strength of this line increased to 124% above that of wild type. Taken together, our results suggest that *OsIRX9/OsIRX9L*, and *OsIRX14*, have similar functions to the *Arabidopsis* *IRX9* and *IRX14* genes, respectively. Furthermore, our expression data indicate that *OsIRX9* and *OsIRX9L* may function in building the xylan backbone in the secondary and primary cell walls, respectively. Our results provide insight into xylan biosynthesis in rice and how expression of a xylan synthesis gene may be modified to increase stem strength.

## Introduction

Plant cells are surrounded by strong walls composed largely of cellulose, matrix polysaccharides, and – in some cell types – lignin. Hemicelluloses and pectin are polysaccharides of the cell wall matrix. Xylans are by far the most abundant matrix polysaccharides of dicot wood and grass cell walls, making it the second most abundant polysaccharide on Earth (Scheller and Ulvskov, 2010). Xylans are a major component of the dietary fiber in cereal grains, and therefore represent a large portion of the human and livestock diet (Ebringerova and Heinze, 2000). The chemical composition of xylans affects the properties of bread making and beer malting (Vinkx and Delcour, 1996). Xylans are also a target for the improvement of feedstocks for the generation of cellulosic biofuels, a currently expensive and inefficient process (Yang and Wyman, 2004; Carroll and Somerville, 2009; Klein-Marcuschamer et al., 2011). Xylans, cellulose, and lignin are important structural components of the plant cell wall. While the down-regulation of the synthesis of xylans (Lee et al., 2011), cellulose (Kokubo et al., 1989, 1991), and lignin (Vanholme et al., 2008) have been shown to decrease the strength of the plant, it is unknown whether the upregulation of secondary wall synthesis genes could increase plant strength. Thus, a greater understanding of xylan biosynthesis may contribute to agriculture, as well as the food and energy industries.

Xylans are structurally diverse, with the substituents on the xylan polymer backbone varying by taxonomy. Xylans of embryophytes have a backbone consisting of β-1,4-linked xylosyl residues. Dicot xylans are commonly substituted with α-(1 → 2)-linked glucuronosyl and 4-*O*-methyl glucuronosyl residues (Ebringerova and Heinze, 2000). Xylans in birch, spruce, and *Arabidopsis* have been found to contain the reducing end oligosaccharide β-d-Xyl*p*-(1 → 4)-β-d-Xyl*p*-(1 → 3)-α-l-Rhap-(1 → 2)-α-d-Gal*p*A-(1 → 4)-d-Xyl*p* (Johansson and Samuelson, 1977; Andersson et al., 1983; Peña et al., 2007) which, interestingly, has not been found in the xylan of grasses. Grass xylans have very few glucuronosyl residues, but are mostly substituted with α-1,2 and α-1,3 arabinosyl residues. Grass xylans are also known to contain other unique chain decorations, including the disaccharide, β-Xyl*p*-(1 → 2)-α-Ara*f*-(1 → 3) (Wende and Fry, 1997; Chiniquy et al., 2012). Another unique feature of grass xylans is the esterification of some arabinosyl residues with ferulic and *p*-coumaric acid.

The majority of genes involved in grass xylan biosynthesis are unknown, despite significant efforts over the past decade to identify the genes involved. In *Arabidopsis*, the *irregular xylem* (*irx*) mutants, named for their collapsed xylem vessels due to secondary cell wall deficiencies, have been useful in elucidating the mechanisms of xylan biosynthesis (Turner and Somerville, 1997). IRX9/IRX9L and IRX14/IRX14L from glycosyltransferase (GT) family 43, and IRX10/IRX10L from GT47 and OsIRX10 are thought to be responsible for elongation of the xylan backbone (Brown et al., 2007, 2009; Geisler-Lee et al., 2007; Persson et al., 2007; Cantarel et al., 2009; Wu et al., 2009; Faik, 2010; Chen et al., 2012). IRX7 (FRA8)/IRX7L (F8H) (from GT47), IRX8 (GAUT12) (from GT8), and PARVUS (from GT8) may be responsible for synthesizing the oligosaccharide found at the reducing end of some dicot and conifer xylans (Brown et al., 2007; Lee et al., 2007b; Liepman et al., 2010; Scheller and Ulvskov, 2010). GXMT1 is a methyltransferase that specifically methylates glucuronosyl residues in xylan to 4-*O*-methyl-glucuronic acid (Urbanowicz et al., 2012). Two other members of the same protein family, the *Arabidopsis* IRX15/IRX15L are essential for xylan deposition in the secondary cell wall (Brown et al., 2011; Jensen et al., 2011) but it is not clear if they are also methyltransferases and what their substrate might be. GUX1, GUX2, and GUX4 (from GT8) add glucuronosyl substitutions to the xylan backbone in *Arabidopsis* (Mortimer et al., 2010; Oikawa et al., 2010; Rennie et al., 2012). Recently the rice *XAT* genes from GT61 were characterized as encoding proteins adding the α-(1 → 3)-arabinosyl substitutions onto the xylan chain (Anders et al., 2012), and rice *XAX1*, also from GT61, was shown to be responsible for adding the xylose residues in Xyl*p*-(1 → 2)-α-Ara*f*-(1 → 3) substitutions (Chiniquy et al., 2012).

Even though there are clear differences in xylan structure between grasses and dicots, it is unknown whether xylan synthesis genes are functionally conserved between *Arabidopsis* and rice. Complementation studies, which involve the heterologous expression of a putative xylan synthesis gene in well-characterized xylan mutants have increased our understanding of xylan synthesis in other plant species. Complementation studies indicated that the poplar *GT43B* gene may be a functionally equivalent ortholog of the *Arabidopsis IRX9* gene (Zhou et al., 2007) and the poplar *GT43C/D* genes are functionally equivalent orthologs to *Arabidopsis IRX14* (Lee et al., 2011). The Poplar *GT47C* and *GT8E/F* are thought to be functionally equivalent orthologs to the *Arabidopsis* *FRA8* and *PARVUS*, respectively (Zhou et al., 2006; Lee et al., 2009).

While the studies mentioned above suggest that IRX9/IRX9L and IRX14/IRX14L are all involved in and essential for synthesis of the xylan backbone in *Arabidopsis* and that IRX10/IRX10L are essential in rice and *Arabidopsis*, it is unclear why three different GTs would be required to make a single transfer reaction, and it is also unclear if xylan biosynthesis would require orthologs of all these proteins in all plant species. A transcriptomic study of psyllium seeds, which are exceptionally rich in xylan, showed high abundance of a transcript corresponding to *IRX10*, but transcripts of genes homologous to *IRX9* and *IRX14* were not detected (Jensen et al., 2011). This would suggest that in this dicot plant, IRX9 and IRX14 might not be required for synthesis of seed xylan. Likewise, a highly active enzyme preparation from wheat capable of synthesizing xylan was purified and immunoprecipitated (Zeng et al., 2010). The enzyme preparation contained orthologs of *IRX10* and *IRX14*, but not of *IRX9*.

To gain a greater understanding of xylan synthesis in rice, we conducted a complementation study of three rice genes that are closely related to the *IRX9*, *IRX9L*, and *IRX14* *Arabidopsis* genes. Here, we demonstrate that the over-expression of *Os07g49370* (*OsIRX9*), *Os01g48440* (*OsIRX9L*), and *Os06g47340* (*OsIRX14*), complemented to varying levels the dwarfed phenotype, irregular xylem cells, decreased xylose, xylosyltransferase (XylT) activity, stem strength, and xylan chain length of the respective *Arabidopsis irregular xylem mutants, irx9* and *irx14*. We also show that *OsIRX9L* was more highly expressed in many developing tissues in wild type rice, with *OsIRX9* expression almost entirely in tissues rich in secondary cell walls – indicating a potential functional differentiation between *IRX9* and *IRX9L* genes. In addition, we show that the over-expression of *OsIRX9* in *irx9* increased the stem strength to above that of wild type plants. Our results provide insight into xylan biosynthesis in rice and demonstrate that expression of a xylan synthesis gene may be modified to increase stem strength.

## Results

### Phenotypic characterization of rice *OsIRX9*, *OsIRX9L*, and *OsIRX14* over-expression lines in the *irx9* and *irx14* *Arabidopsis* mutants

**Figure 1:**
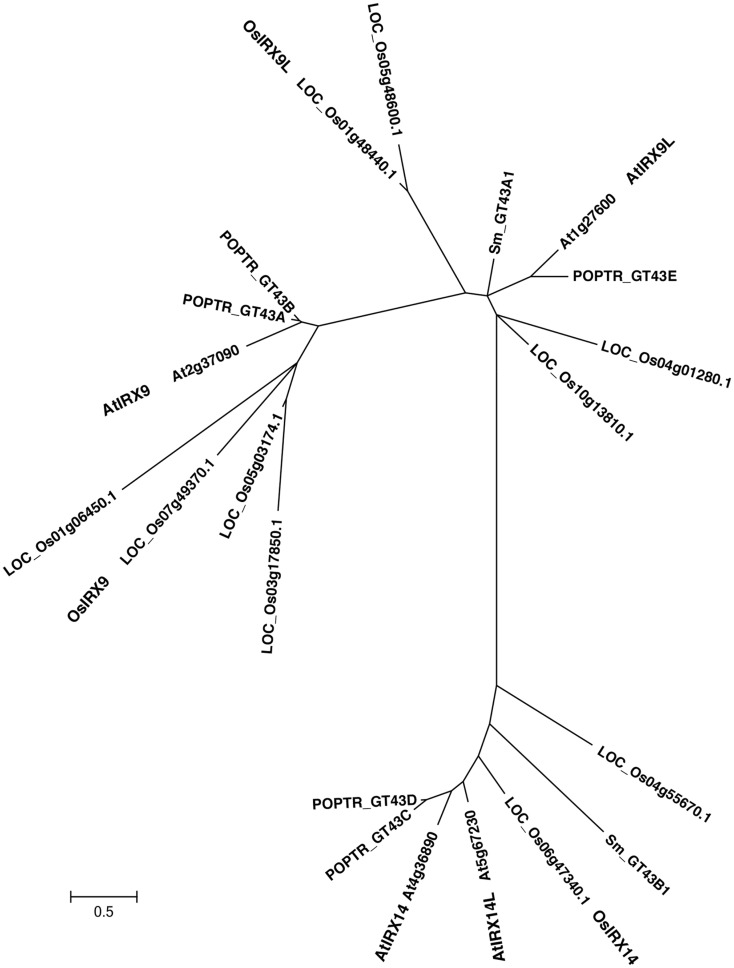
**Phylogenetic tree of glycosyltransferase family 43 including genes from rice, *Arabidopsis*, Poplar, *Selaginella*, and *Physcomitrella* genomes**. The evolutionary relationships were inferred using the Neighbor-Joining method. The optimal tree with the sum of branch length = 16.96637902 is shown. The tree is drawn to scale with branch lengths in the same units as those of the evolutionary distances used to infer the phylogenetic tree. The evolutionary distances were computed using the JTT matrix-based method and are in the units of the number of amino acid substitutions per site. The rate variation among sites was modeled with a gamma distribution (shape parameter = 1). Evolutionary analyses were conducted in MEGA5.

**Figure 2:**
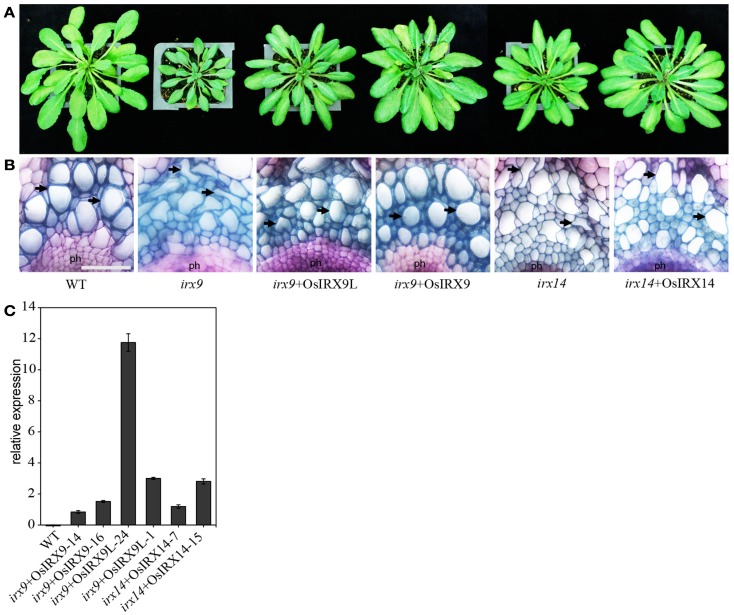
**Restoration of (A) plant size and (B) irregular xylem vessel phenotype in *irx9* and *irx14* mutant plants by over-expression of rice genes**. Stem cross sections were stained with toluidine blue. Phloem (ph) and xylem vessels (arrows) are indicated. Scale bar = 50 μm. **(C)** Relative expression of each rice gene in the complemented *Arabidopsis* lines. The relative expression levels were examined for 10 lines in each construct. Two lines (shown) with the highest expression for each construct were chosen for further analysis. Error bars represent SD of three biological replicates.

**Figure A1:**
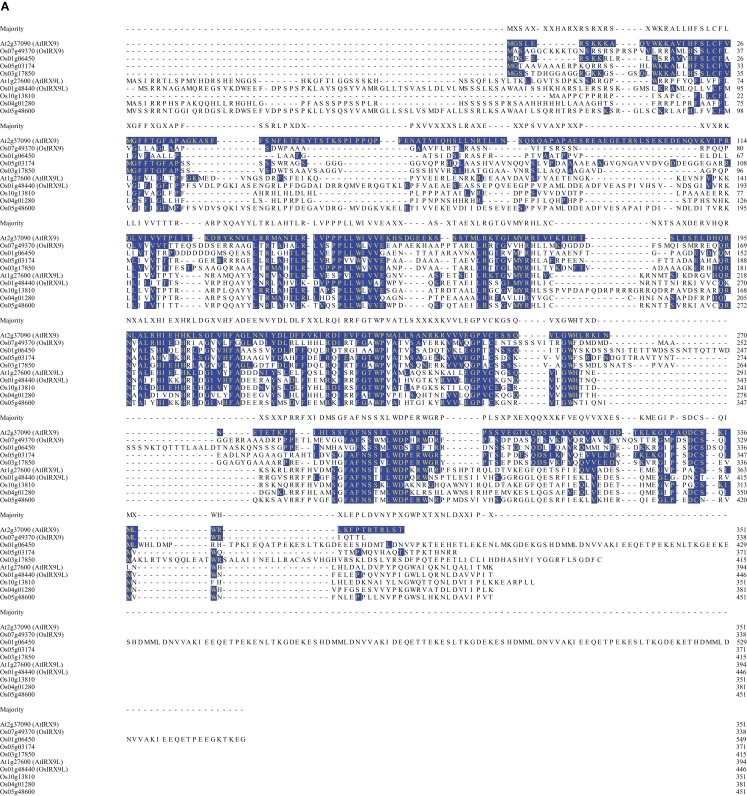

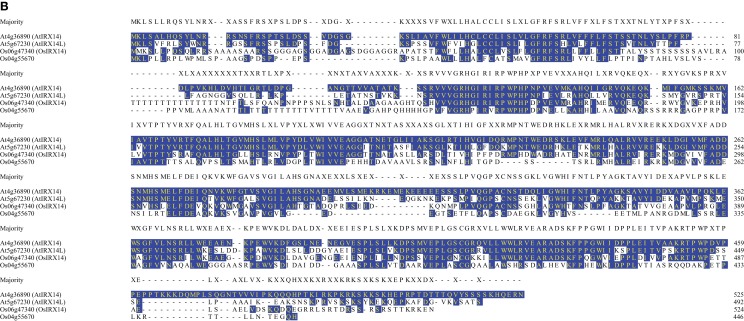
**Protein sequence alignments for (A) AtIRX9 and AtIRX9L, and (B) AtIRX14 and the respective closely related rice genes**. Amino acids with blue shading and yellow lettering are 100% conserved compared to AtIRX9 and AtIRX14, respectively.

To determine the functional equivalence of *Os07g49370*, *Os01g48440*, and *Os06g47340*, to the respective closely related *Arabidopsis* genes (Figure 1 and Figure A1 in Appendix), *IRX9*, *IRX9L*, and *IRX14*, respectively, we over-expressed the three rice genes, hereafter referred to as *OsIRX9*, *OsIRX9L*, and *OsIRX14* in the *Arabidopsis irx9* and *irx14* mutant plants. Expression levels were evaluated in 10 independently transformed lines using rice gene specific primers. Two lines from each transformant with the highest expression were selected for further characterization (Figure 2C). Complementation of the *Arabidopsis* *irx9* mutant with rice OsIRX9 (*irx9* + OsIRX9) resulted in phenotypes similar to the Columbia (Col-0) wild type control at 5-weeks post germination. Microscopy of stem cross sections indicated the absence of irregular xylem cells (Figures 2A,B). Similarly, complementation of OsIRX14 in the *Arabidopsis* *irx14* mutant (*irx14* + OsIRX14) resulted in a similar plant size and regularity of the xylem cells to that of the wild type control plant. The *irx9* + OsIRX9L appeared to have an intermediate level of complementation between wild type and *irx9* plants both in terms of plant height and xylem vessel appearance (Figures 2A,B).

### Biochemical analysis and stem strength measurements

**Figure 3:**
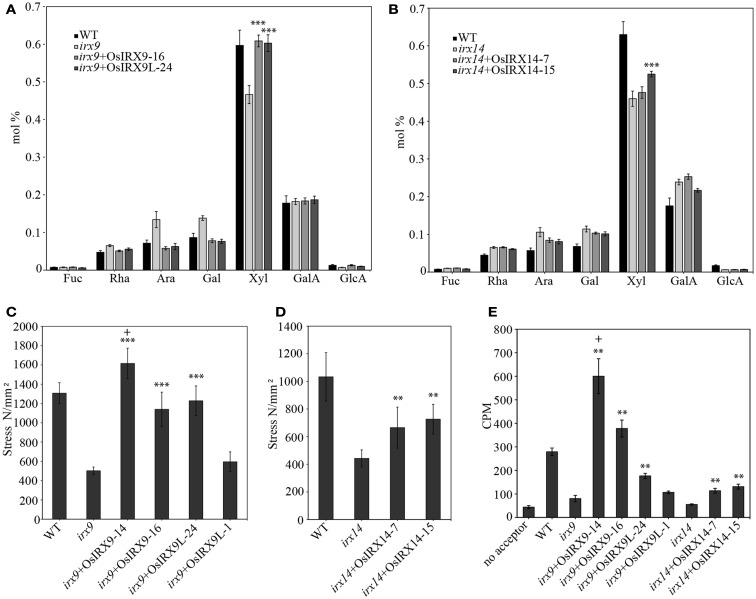
**Biochemical and mechanical strength analyses of 5-week-old stems in complemented plants**. Cell wall composition analysis shows restoration of xylose deficiency by over-expression of **(A)** rice *OsIRX9* and *OsIRX9L* genes. Error bars represent SD with at least 10 biological replicates. **(B)** rice *OsIRX14* genes. Error bars represent SD with at least five biological replicates. Restoration of stem strength in over-expression of **(C)** rice *OsIRX9* and *OsIRX9L* genes and **(D)** rice *OsIRX14* genes. Error bars represent SD with at least eight biological replicates. **(E)** Restoration in xylosyltransferase activity of *irx9* and *irx14* mutant plants by over-expression of rice genes. The two OsIRX9 lines had xylosyltransferase activity that exceeded that of wild type. Error bars represent SD with three biological replicates. Key: significantly different from respective mutant background by *t* test *p < 0.05, **p < 0.01, ***p < 0.001; significantly different from WT by *t* test:+*p* < 0.05.

**Figure 4:**
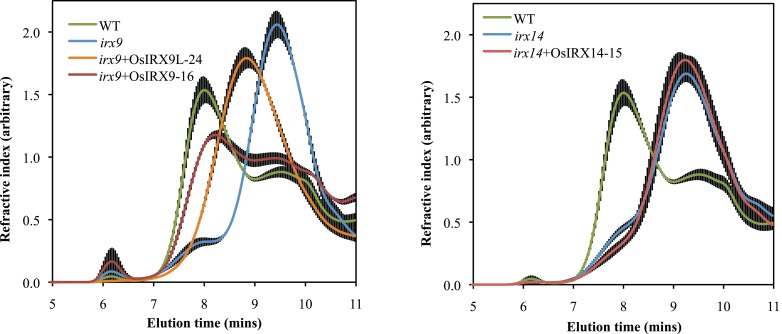
**Size-exclusion chromatography (SEC) of mutant, wild type, and complemented lines**. Length of xylan chain is measured by elution time (min). Results indicate that the OsIRX9L line has a xylan length that is intermediate between that of wild type and *irx9*; The OsIRX9 complemented line has a xylan length comparable to wild type; The OsIRX14 complemented line appears to have a xylan chain length like the *irx14* mutant. Equal amounts of carbohydrate were loaded onto the column. Error bars represent SD with at least three biological replicates.

The *irx9* and *irx14* mutant plants have stems with a decreased xylose content and residual xylan with a significantly lower molecular mass (Brown et al., 2007; Peña et al., 2007). To determine the level of complementation in terms of xylose content in the rice over-expression lines, we prepared cell wall alcohol insoluble residue (AIR), enzymatically removed starch, and acid hydrolyzed the non-cellulosic polysaccharides. The released monosaccharides were separated and quantified by high performance anion exchange chromatography with electrochemical detection (HPAEC-PAD). The *irx9* + OsIRX9 and *irx9* + OsIRX9L xylose contents in stems recovered to those of wild types (Figure 3A) (both with a p value of less than 0.001 using a *t* test). The *irx14* + OsIRX14 xylose content reached an intermediate level of complementation but was significantly above that of *irx14* (Figure 3B) (*t* test: *p* < 0.001). To determine if the increase in xylose content also correlated with the length of the xylan chains being restored, we used size-exclusion chromatography (SEC) to measure the size distribution of the xylans in the mutants, wild type, and complemented lines (Figure 4). We found that the *irx9* + OsIRX9L line reached an intermediate level of complementation in terms of xylan chain length, with the *irx9* + OsIRX9 line having a chain length comparable to wild type. Interestingly, the *irx14* + OsIRX14 xylan chain length was comparable to the *irx14* mutant.

The decrease in xylan in the secondary walls of the *irx9* and *irx14* mutant plants results in a lower stem strength. To determine if the rice genes complemented the stem strength of the *Arabidopsis* mutants, we measured the stem strength in the rice over-expression plants and found that one *irx9* + OsIRX9 line 14 demonstrated a stem strength that was 124% that of wild type (Figure 3C) (*t* test: *p* < 0.05). The data from multiple lines from each construct are shown – and the *irx9* + OsIRX9 line 16 had a stem strength that was 87% that of wild type, but this still is a significant improvement in strength considering that the uncomplemented *irx9* mutant had a stem strength of 38% that of wild type. The two *irx9* + OsIRX9L lines also showed improvement in stem strength that was 94 and 46% that of wild type – the first line complementing far better. The *irx14* mutant stems had a breaking strength that was slightly higher than *irx9* at 43% that of wild type (Figure 3C). The two complemented *irx14* + OsIRX14 lines demonstrated a stem strength that was 64 and 70% that of wild type (Figure 3D) (*t* test: *p* < 0.01).

To determine the level of XylT activity in the stems of the complemented plants, microsomes were extracted from 5-week-old stems for each plant line, and ^14^C-xylose incorporation onto a xylohexaose acceptor in the presence of UDP-^14^C-xylose was measured (Figure 3E). As a reference, the *irx9* and *irx14* plant stems demonstrated XylT activities that were 29 and 20%, respectively, to that of wild type. Notably, the *irx9* + OsIRX9 line 14 demonstrated a XylT activity that was more than twice that of wild type (*t* test: *p* < 0.05). The second *irx9* + OsIRX9 line was 135% that of wild type. The two *irx9* + OsIRX9L lines were 63% (*t* test: *p* < 0.01) and 38% that of wild type, and the two *irx14* + OsIRX14 lines were 41 and 47% that of wild type (*t* test: *p* < 0.01). Overall, in terms of XylT activity, all lines showed a level of recovery from the *irx9* and *irx14* mutants, but only the *irx9* + OsIRX9 lines exceeded the XylT activity of the wild type.

### Tissue specific expression of *OsIRX9*, *OsIRX9L*, and *OsIRX14* genes in wild type rice

**Figure 5:**
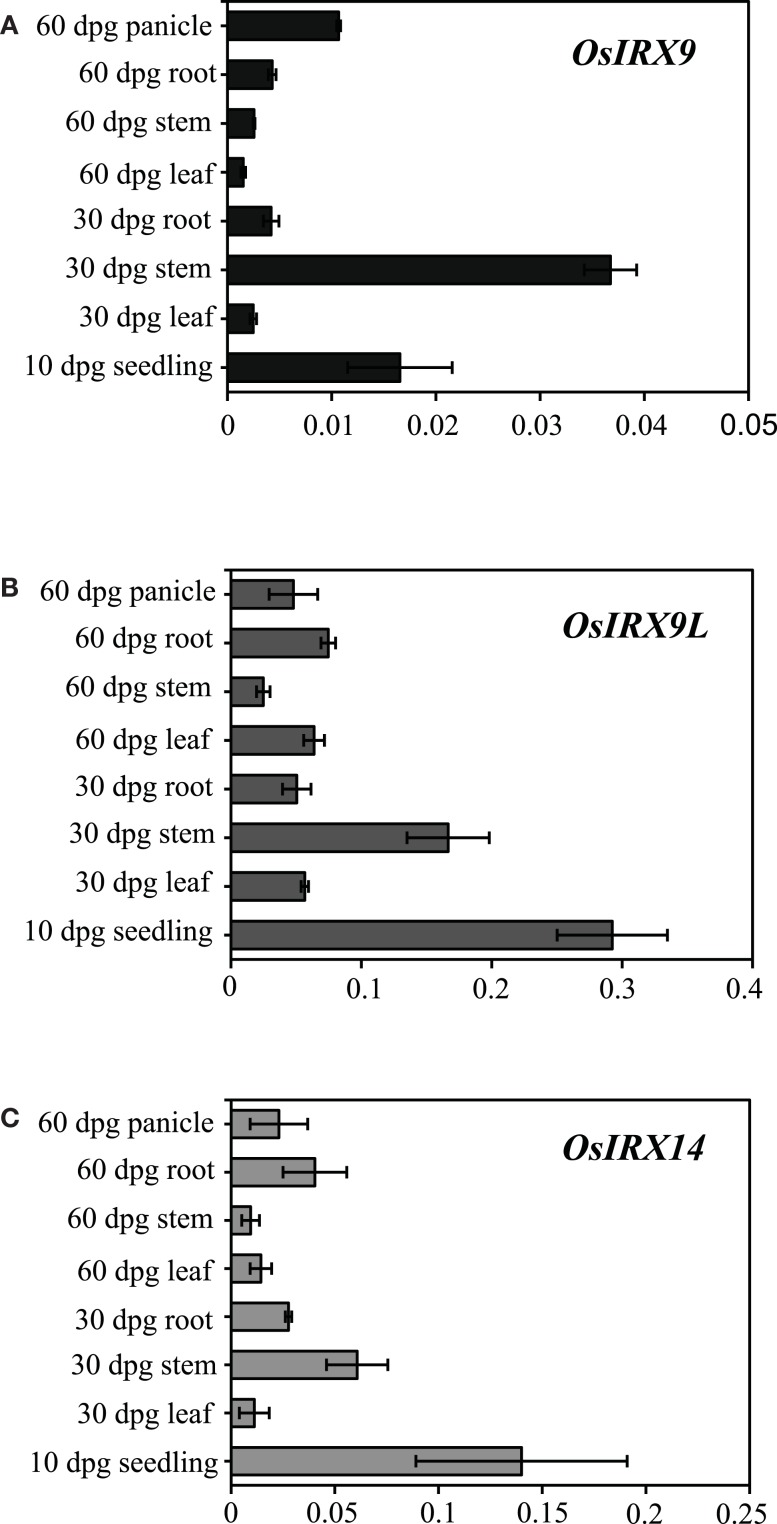
**Relative expression in wild type rice plants measured by qPCR with rice gene specific primers of (A) *OsIRX9* (*Os07g49370*) (B) *OsIRX9L* (*Os01g48440*) and (C) *OsIRX14* (*Os06g47340*) in various tissues at 10, 30, and 60 days post germination (dpg)**. Error bar represent SD with three biological replicates.

While dicots have abundant xylan in secondary walls and very low amounts in the primary walls, grass xylan is abundant in both primary and secondary cell walls (Vogel, 2008). Accordingly, *IRX9* and *IRX14* genes in *Arabidopsis* plants are primarily expressed in cells undergoing secondary wall synthesis (Peña et al., 2007). To analyze the potentially different expression patterns in rice, we used quantitative PCR with gene specific primers to determine the tissue specific expression of the *OsIRX9*, *OsIRX9L*, and *OsIRX14* genes in wild type rice plants (Figure 5). Overall, *OsIRX9* is expressed at a much lower level than *OsIRX9L*, a difference from what has been reported in *Arabidopsis* (Schmid et al., 2005). Similar to *Arabidopsis* expression, rice *OsIRX9L* is expressed at moderate levels in many tissues, including leaves and roots, whereas *OsIRX9* is most prominently expressed in the stem. All three genes have the highest level of expression in the 10 days post germination (dpg) seedling and in the 30 dpg stem tissue.

## Discussion

### The putative functional divergence of *IRX9* and *IRX9L*

*IRX9*, *IRX9L*, and *IRX14* are all members of the GT43 family, and are essential for elongation of the xylan backbone, a process that is expected to be conserved between dicots and commelinid monocots. Accordingly, our results have demonstrated that *OsIRX9*, *OsIRX9L*, and *OsIRX14* have overlapping functions with their *Arabidopsis* counterparts in terms of plant phenotypes (Figure 2A), presence of irregular xylem cells (Figure 2B), xylose content of stems (Figures 3A,B), stem strength (Figures 3C,D), xylan chain length (Figure 4), and XylT activity (Figure 3E). Interestingly, we found that *OsIRX14* was able to complement *irx14* to an intermediate level in terms of xylose content of stems and XylT activity, but not in terms of xylan chain length, indicating that the complemented line was making more xylan chains. We found that *OsIRX9L* was not able to complement the *Arabidopsis irx9* mutant plants as well as the *OsIRX9* gene. These findings are consistent with (Lee et al., 2010) who found that the *Arabidopsis IRX9L* gene in the *irx9* background had an intermediate level of complementation in terms of stem breaking strength and stem XylT activity. In contrast, Wu et al. (2010) concluded that IRX9 and IRX9L were essentially identical in their ability to complement *irx9*/*irx9L* mutants, but they based this on appearance of the plants rather than biochemical characterization. We also found that *OsIRX9L* was more highly expressed in many developing tissues in wild type rice, including leaves, with *OsIRX9* expression almost entirely in tissues rich in secondary cell walls (Figure 5). As seen in the phylogenetic tree in Figure 1, both *Selaginella* and *Physcomitrella*, basal plant species from Lycopodiophyta and Bryophyta, respectively, have *IRX9L* and *IRX14* orthologs, but no *IRX9* ortholog (Kulkarni et al., 2011; Harholt et al., 2012). The vascular tissues in Lycopodiophytes have cells with thickened walls, but several types of evidence suggest that the vasculature of Lycopodiophytes has a different evolutionary origin than in the Euphyllophytes (Harholt et al., 2012).

Dicots, including *Arabidopsis*, have very little xylan in the primary cell walls, with most xylan deposition present in the secondary cell walls. This could explain why *IRX9* is more highly expressed in *Arabidopsis* than *IRX9L*, and also why both genes are expressed in rice, which has an abundance of xylan in both primary and secondary cell walls, with xylan playing an important role in young tissue, such as the rapidly expanding cells of the seedling. Taken together, our data suggests a functional divergence of *IRX9* and *IRX9L*, with *IRX9* being important for biosynthesis of xylans in the secondary cell wall and *IRX9L* being important in the primary cell wall. Our results also suggest that *IRX9L* may play more of an important role in species where there is more xylan present in the primary cell walls, such as the grasses. Since the biochemical function of plant GT43 proteins is not known, future studies will be needed to elucidate possible functional divergence between members of the IRX9 and IRX9L clades. A notable structural difference is the lack of a conserved DXD motif in the IRX9 clade, while this motif is highly conserved in the IRX9L clade (Figure 1 in Appendix). The DXD motif is generally present in GTs with a GT-A fold such as members of GT43, and is required for binding of the divalent metal ion that coordinates the nucleotide sugar substrate and facilitates catalysis (Breton et al., 2012).

### Do *IRX9* and *IRX14* operate non-redundantly in a protein complex for β-(1,4)-xylan xylosyltransferase activity?

*Arabidopsis* IRX9 and IRX14 function non-redundantly in building the β-(1,4)-xylan backbone (Lee et al., 2010; Wu et al., 2010), but neither of these proteins, nor IRX10 has been biochemically purified and retained their XylT activity. This lends support to the hypothesis that they operate together in a protein complex. In support of these proteins operating in a complex, Zeng et al. (2010) used TaGT43-4, which is closely related to the *Arabidopsis* IRX14 protein, to co-immunoprecipitate a protein complex in wheat that had XylT, AraT, and GlcAT activities that worked in a cooperative manner. Lee et al. (2012) heterologously expressed *IRX9* and *IRX14* in tobacco cells and demonstrated a substantial increase in the XylT activity as compared to plants expressing either *IRX9* or *IRX14* alone, lending support to the hypothesis that *IRX9* and *IRX14* operate cooperatively. We found an increase of XylT activity that was over twice that of wild type *Arabidopsis* stems when the rice *OsIRX9* gene alone was over-expressed in *irx9* plants (Figure 3E). These results are consistent with studies in which an increase over wild type stem XylT activity was reported in *irx9* plants with over-expression of *IRX9* (Lee et al., 2010, 2011). This could indicate either that IRX9 was the limiting protein in the protein complex or that IRX9 can operate without a protein complex in building the β-(1,4)-xylan backbone. It is also possible that these two proteins are biochemically inactive, serving a structural role for the IRX10 protein, which is known to play a role in building the xylan backbone (Brown et al., 2009). This is similar to the proposed function of the GAUT7 anchoring GAUT1 in a protein complex for pectin biosynthesis (Atmodjo et al., 2011). In agreement with this hypothesis, a study of the ESTs derived from the *Psyllium* (*Platago ovata*) seed mucilaginous layer, which is rich in xylan, detected abundant amounts of transcript for *IRX10*, and very little if any for *IRX9* or *IRX14*, indicating that IRX10 is sufficient to synthesize xylan in that tissue (Jensen et al., 2011). More work must be completed to better understand the mechanism of xylan chain synthesis and how the IRX9, IRX14, and IRX10 proteins operate in building the xylan backbone.

### Over-expression of *OsIRX9* in *Arabidopsis* leads to an increase in stem strength

The secondary cell walls of dicots are almost entirely composed of cellulose, lignin, and xylan (Vogel, 2008). The down-regulation of the synthesis of xylan (Lee et al., 2011), cellulose (Kokubo et al., 1989, 1991), and lignin (Vanholme et al., 2008) have been demonstrated to decrease the strength of plant secondary cell walls. Our results demonstrate that stem strength is significantly increased to 124% that of wild type with the heterologous expression of the *OsIRX9* gene (Figure 3C). This could be due to reinforcement of the secondary cell walls in the vessel elements, although cross sections showed no discernable increase in vessel wall thickness (Figure 2B). Interestingly, Lee et al. (2011) found that the over-expression of the poplar GT43A, B, and E genes in *Arabidopsis irx9* plants rescued the stem strength phenotype to the level of wild type, but not above that level. Likewise, the over-expression of *AtIRX9* in *Arabidopsis irx9* plants did not lead to an increase in stem strength over that of wild type plants (Lee et al., 2010). It is unclear why over-expression of a rice xylan synthase gene, but not its poplar or *Arabidopsis* orthologs, in *Arabidopsis* would increase stem strength. However, as plant stem lodging is a significant cause of crop losses worldwide (Berry et al., 2004; Hall et al., 2009; Ma, 2009), the finding that heterologous over-expression of a rice xylan synthesis gene can increase the strength of a plant stems has important implications for crop plant biotechnology.

In conclusion, rice *OsIRX9*, *OsIRX9L*, and *OsIRX14* have overlapping functions with the *Arabidopsis* counterparts. We also show that *OsIRX9L* is more highly expressed in many developing tissues in wild type rice, with *OsIRX9* expression almost entirely in tissues rich in secondary cell walls – indicating a potential functional differentiation between *IRX9* and *IRX9L* genes. In addition, we have found that heterologous over-expression of rice *OsIRX9* in *Arabidopsis* *irx9* plants increases the stem strength beyond that of wild type.

## Materials and Methods

### Plant growth and plant transformations

*Arabidopsis thaliana* accession Columbia-0 (Col-0) was obtained from the *Arabidopsis* Biological Resource Center (ABRC^1^). T-DNA insertion mutants (*irx9*, Salk_058238; *irx14*, Salk_038212) were localized in the SIGnAL Salk^2^ collections and obtained from the ABRC. Homozygous plants were identified by PCR with gene specific primers (Table 1). Seeds were germinated and seedlings then grown on soil (PRO-MIX, Premier Horticulture Inc., Quakertown, PA, USA) in a growth chamber under short-day light conditions (10 h photoperiod, 120 μmol m^−2^ s^−1^, at 22°C and 60% RH/14 h of dark at 22°C and 60% RH). After 3 weeks, plants were transferred to long-day conditions (16 h photoperiod/8 h dark; otherwise as above). *Arabidopsis* plants were transformed using *Agrobacterium tumefaciens* GV 3101 pmp90 via the floral dip method (Clough and Bent, 1998). For BASTA selection, seeds were germinated on soil as described above and sprayed every 2 days for a total of five times with a glufosinate-ammonium (Crescent Chemical Company, Islandia, NY, USA) solution (40 mg/ml). Resistant plants were transferred to new pots and further grown, as described above.

**Table 1:** **List of primers used for gene cloning and quantitative PCR**.

Primer name	Orientation	Sequence (5′ to 3′)	Target
Os07g49370 F	Sense	CACCATGGCGTCGGCAGGTGGCTGCAAG	Os07g49370
Os07g49370 R	Antisense	CTAGAGCGTAGTTTGGATGCG	Os07g49370
Os01g48440 F	Sense	CACCATGTCCCGAAGGAATGCCGGGGCA	Os01g48440
Os01g48440 R	Antisense	TTATGTTATTGGCACAACAGCATC	Os01g48440
Os06g47340 F	Sense	CACCATGATGAAGTCGCTGCTGCCG	Os06g47340
Os06g47340 R	Antisense	TCAGTTCTCCTTCCGCTTTGTGGT	Os06g47340
Os07g49370 qPCR F	Sense	CTCCGGAGACGTTAATGGAAGT	qRT PCR amplicon Os07g49370
Os07g49370 qPCR R	Antisense	CTGCACGAACTTCACTGATTCC	qRT PCR amplicon Os07g49370
Os01g48440 qPCR F	Sense	GTATAGTGCATTTCGCTGATGAAG	qRT PCR amplicon Os01g48440
Os01g48440 qPCR R	Antisense	TTCTAGAACCACTCTGTACTTTGTCC	qRT PCR amplicon Os01g48440
Os06g47340 qPCR F	Sense	GTCACGCAACCGAGAATCGTAT	qRT PCR amplicon Os06g47340
Os06g47340 qPCR R	Antisense	AGCTATGAACATTGCTGTCATCC	qRT PCR amplicon Os06g47340
UBQ 10 F	Sense	GGCCTTGTATAATCCCTGATGAATAAG	qRT PCR reference gene
UBQ 10 R	Antisense	AAAGAGATAACAGGAACGGAAACATAGT	qRT PCR reference gene
IRX9F	Sense	GCTGGTAAGGCCTCATTTTTC	Genotyping *irx9*, Salk_058238
IRX9R	Antisense	AACTTACCAACCCACCCATTC	Genotyping *irx9*, Salk_058238
IRX14F	Sense	AACGACACGTGTACCTCCTTG	Genotyping *irx14*, Salk_ 038212
IRX14R	Antisense	AACATCACAATCCCATCAAGC	Genotyping *irx14*, Salk_ 038212
LBa1	Sense	TGGTTCACGTAGTGGGCCATCG	Left border primer of SALK lines

### Gene cloning

The cDNAs for *Os07g49370*, *Os01g48440*, and *Os06g47340* were amplified by PCR using gene specific primers (Table 1) from first strand cDNA made from pooled rice samples. Coding sequences for genes were cloned using Gateway technology (Invitrogen) and Gateway-compatible primers (Table 1), as follows: PCR reaction products were gel-purified using the MinElute Gel extraction kit (Qiagen, Valencia, CA, USA) and used for recombination reactions into pENTR-D-TOPO cloning (Invitrogen), then recombination into the destination vector pEarleyGate 101 (Earley et al., 2006) using LR clonase enzyme mix (Invitrogen).

### Expression analysis

Total RNA was extracted using the RNeasy plant mini kit (Qiagen, Valencia, CA, USA) following the manufacturer’s instructions. RNA preparations were treated with DNase1 (Qiagen, Valencia, CA, USA) to remove traces of DNA contamination. One microgram of RNA was used for reverse transcription with the Transcriptor high fidelity cDNA synthesis kit (Roche) and oligo dT primers. After synthesis, the cDNA reaction was diluted four times in RNAse-free water, and 2 μl was used for PCR using the Fast SYBR Green master mix (Applied Biosystems, Carlsbad, CA, USA) and gene specific primers in a Step ONE plus QPCR machine (Applied Biosystems, Carlsbad, CA, USA). Primers for QPCR are listed in Table 1. QPCR results were normalized to the internal ubiquitin control and is presented as relative expression calculated according to Hellemans et al. (2007).

### Microscopy

Stems from 6-week-old plants were sectioned directly above the second internode. The stems were embedded in 7% agarose and sectioned (60 μm) using a Leica VT1000S vibratome, as described (Manabe et al., 2011). They were stained with a 0.1% toluidine blue solution, and imaged on a Leica MZ16F fluorescence stereomicroscope under bright field (40×).

### Stem strength

Ultimate stress was measured using an in-house tensile testing instrument (Vega-Sanchez and Ronald, 2010; Varanasi et al., 2012). The 5-week-old plant stems were cut at the seed using a razor blade in one stroke without any damage to the stem. The diameter of the stem at the second internode and its total length were measured. The stem segment was then glued on to the sample holders using hot glue (Stanley DualMelt). Only 5 mm of the stem remained unglued between the sample holders. The sample holders were then screwed on to the apparatus and the tensile strength measurements were taken at room temperature. The sample holders consisted of a support system for the unglued portion of the stem to prevent it from damage during the holder installation. The support was removed from the holder just before starting the analysis. Stress was calculated as a ratio of the force and cross-sectional area of the stem.

### Cell wall isolation and monosaccharide composition analysis

For *Arabidopsis* transformants, 6-week-old primary stem tissue was collected, frozen in liquid nitrogen, and freeze-dried overnight using a lyophilizer. AIR preparation and destarching was performed according to methods described by Yin et al. (2011). For monosaccharide composition analysis, 5 mg was hydrolyzed in 2 M trifluoroacetic acid at 120°C for 1 h. The released monosaccharides were separated by HPAEC on a Dionex ICS3000 system (Sunnyvale, CA, USA) equipped with a pulsed amperometric detector (PAD) as described by Harholt et al. (2006).

### Microsomal extraction of *Arabidopsis* stems

For protein isolation, 6-week-old whole stems were flash frozen, ground in a mortar and pestle in 15 mL of buffer (50 mM HEPES pH 7.0, 400 mM sucrose, 1 mM PMSF, 1% w/v PVPP, Protease Inhibitor Cocktail). This was then filtered through Miracloth mesh and centrifuged at 3,000 × *g* for 10 min. The supernatant was then centrifuged at 50,000 × *g* at 4°C for 1 h. (Beckman Ultracentrifuge). The pellets containing the microsomes were resuspended in a 50 mM HEPES pH 7.0, 400 mM sucrose buffer and stored at −80°C. Protein concentration was determined using the Bradford method.

### Xylosyltransferase activity assay

Microsomal activity assays were based on the protocol described by (Lee et al., 2007a). Microsomes corresponding to 40 μg protein were incubated with 50 mM HEPES-KOH, pH 6.8, 400 mM sucrose, 5 mM MnCl_2_, 1 mM DTT, 0.5% Triton X-100, 400 μM xylohexaose, 3.7 μM UDP-[^14^C]Xylose (740 Bq per reaction; American Radiolabeled Chemicals, Inc., St. Louis, MO, USA) in a total reaction volume of 50 μL. After incubation at 24°C for 3 h, reaction was stopped by adding 5 μl termination buffer (0.3 M acetic acid containing 20 mM EGTA). The supernatant was spotted onto Whatman 3 mm chromatography paper (Whatman, Kent, UK), which was then developed in 95% EtOH/1 M ammonium acetate, 2:1 (v/v) as the solvent. The radiolabeled xylooligosaccharides are retained at the original spot, which was cut out and resuspended in 1 ml 100 mM NaOH, an equal volume of scintillation fluid (Ecoscint XR, National Diagnostics, Atlanta, GA, USA) was added, and the amount of activity was determined using scintillation counter set to measure ^14^C counts for 2 min (Beckman LS 6500).

### Size-exclusion chromatography

AIR was extracted over night in 400 μL/mg AIR of 1 M NaOH with 1% v/v NaBH_4_. Insoluble material was removed by centrifugation at 13,000 × *g* and the supernatant was transferred to new tubes, neutralized with HCl, and filtered through 0.45 μm filter. Molecular mass distribution of the extract was determined using a Viscotek GPC Max system equipped with a TDA 305 detector (Malvern, UK). The column used for separation was a Shodex GS-520 equipped with an Ashahipak GS-520 HQ precolumn (Shodex, New York, NY, USA). The eluent was 50 mM NH_3_COOH, run at 0.5 mL/min. Molecular masses were estimated using dextran standards (Fluka). The presence of xylan in the extract was confirmed by enzymatic degradation using 0,1 U/mL extract of xylanase M4 (Megazyme, Ireland) in 100 mM acetate buffer, pH 4.5, overnight. The degraded sample was rerun on the Viscotek as above and the molecular mass distribution compared to the undigested sample.

### Construction of GT43 phylogeny

The GT43 tree was constructed using the Neighbor-Joining method (Saitou and Nei, 1987). The optimal tree with the sum of branch length = 16.96637902 is shown. The tree is drawn to scale, with branch lengths in the same units as those of the evolutionary distances used to infer the phylogenetic tree. The evolutionary distances were computed using the JTT matrix-based method (Jones et al., 1992) and are in the units of the number of amino acid substitutions per site. The rate variation among sites was modeled with a gamma distribution (shape parameter = 1). The analysis involved 21 amino acid sequences. All ambiguous positions were removed from each sequence pair. There was a total of 796 positions in the final dataset. Evolutionary analyses were conducted in MEGA5 (Tamura et al., 2011).

## Conflict of Interest Statement

The authors declare that the research was conducted in the absence of any commercial or financial relationships that could be construed as a potential conflict of interest.

## Footnotes

^1^http://abrc.osu.edu/

^2^http://signal.salk.edu/
